# Supplementary figures and images for: Retrotransposon Insertion Polymorphisms (RIPs) in Pig Coat Color Candidate Genes
Source: Animals (Basel). 2022 Apr 8;12(8):969. doi: 10.3390/ani12080969 (PMC9031378; doi:10.3390/ani12080969)

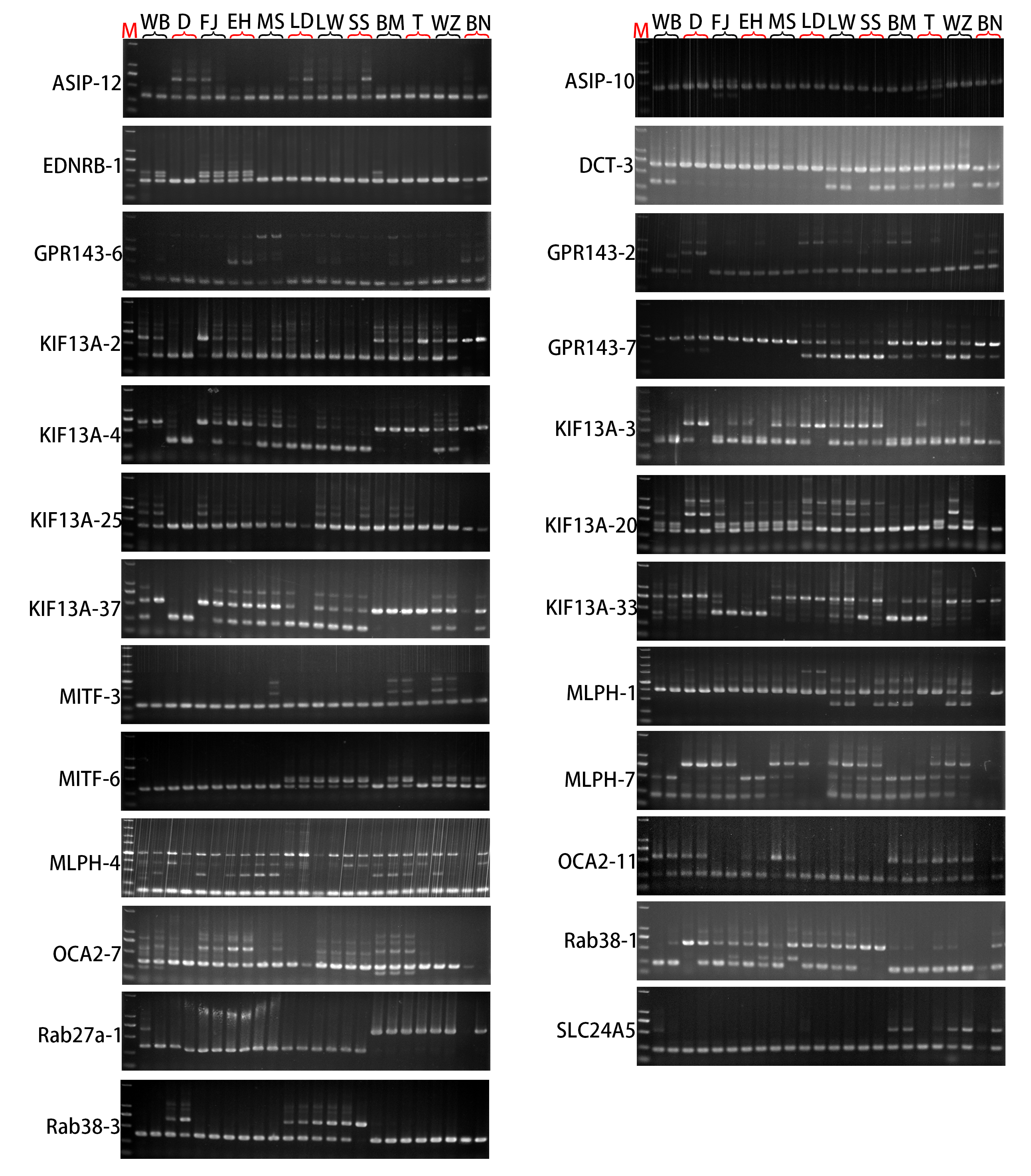

Supplement: Supplementary file 1 [file animals-12-00969-s001.zip › Figure S1 DNA screening results of color genes SVs in pigs_1_.tif]

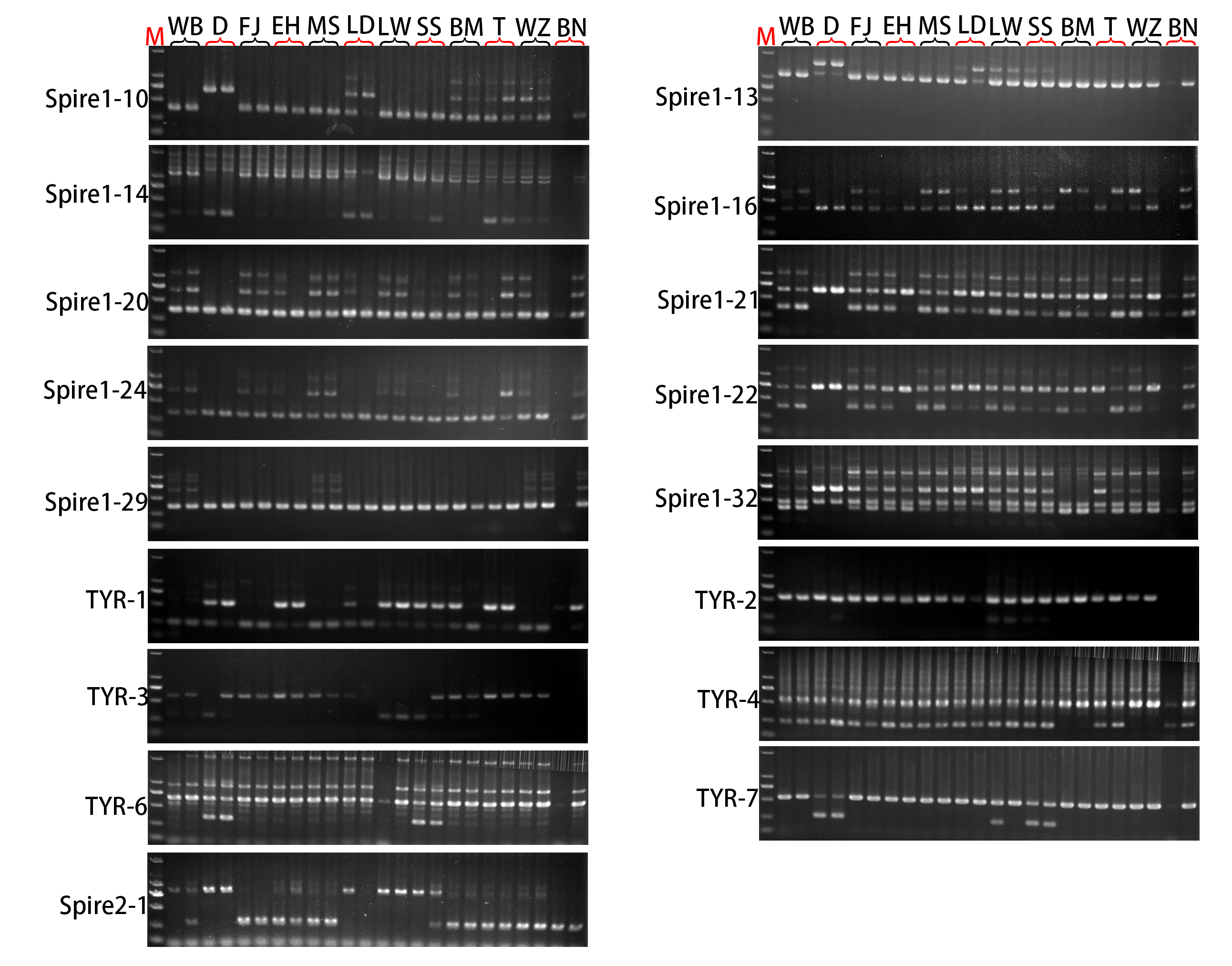

Supplement: Supplementary file 1 [file animals-12-00969-s001.zip › Figure S2 DNA screening results of color genes SVs in pigs_picture_2_.tif]
